# Inferring Evolutionary Timescales without Independent Timing Information: An Assessment of “Universal” Insect Rates to Calibrate a Collembola (Hexapoda) Molecular Clock

**DOI:** 10.3390/genes11101172

**Published:** 2020-10-07

**Authors:** Aron D. Katz

**Affiliations:** 1 Engineer Research Development Center, 2902 Newmark Dr., Champaign, IL 61826, USA; aronkatz@illinois.edu; 2 Department of Entomology, University of Illinois at Urbana-Champaign, 320 Morrill Hall, 505 South Goodwin Ave., Urbana, IL 61801, USA; 3 Illinois Natural History Survey, Prairie Research Institute, University of Illinois at Urbana-Champaign, 1816 South Oak Street, Champaign, IL 61820, USA

**Keywords:** cryptic diversity, cytochrome oxidase subunit I (COI), molecular evolution, phylogeny, rate constancy, relative rates, springtails, substitution saturation

## Abstract

Previous estimates of nucleotide substitution rates are routinely applied as secondary or “universal” molecular clock calibrations for estimating evolutionary timescales in groups that lack independent timing information. A major limitation of this approach is that rates can vary considerably among taxonomic groups, but the assumption of rate constancy is rarely evaluated prior to using secondary rate calibrations. Here I evaluate whether an insect mitochondrial DNA clock is appropriate for estimating timescales in Collembola—a group of insect-like arthropods characterized by high levels of cryptic diversity. Relative rates of substitution in cytochrome oxidase subunit 1 (COI) were inferred via Bayesian analysis across a topologically constrained Hexapod phylogeny using a relaxed molecular clock model. Rates for Collembola did not differ significantly from the average rate or from the rates estimated for most other groups (25 of 30), suggesting that (1) their apparent cryptic diversity cannot be explained by accelerated rates of molecular evolution and (2) clocks calibrated using “universal” insect rates may be appropriate for estimating evolutionary timescales in this group. However, of the 31 groups investigated, 10 had rates that deviated significantly from the average (6 higher, 4 lower), underscoring the need for caution and careful consideration when applying secondary insect rate calibrations. Lastly, this study exemplifies a relatively simple approach for evaluating rate constancy within a taxonomic group to determine whether the use of secondary rates are appropriate for molecular clock calibrations.

## 1. Introduction

The concept of the molecular clock has revolutionized the field of evolutionary biology by providing a foundation for evaluating the tempo of biological processes and mechanisms shaping patterns of biodiversity [1]. Molecular clocks have been widely implemented to estimate divergence times, determine evolutionary rates, and to assess biogeographic hypotheses, but in practice, this powerful statistical tool requires independent information to calibrate rates and timescales into units of absolute time [2]. The most common approach for calibrating molecular clocks is to constrain the minimum age of phylogenetic relationships to dates derived from the fossil record [3] or to the timing of biogeographic events associated with lineage divergence [4]. However, for most small, soft-bodied organisms, informative fossils for calibration are generally unavailable. Furthermore, the use of biogeographic calibrations requires strong assumptions regarding the connection between phylogenetic and biogeographic events, as well as the age of the biogeographic event itself [5]—information that is often unreliable at best and unavailable at worst. In cases where informative fossil and biogeographic information are unavailable, secondary or “universal” substitution rates derived from previous studies of related taxa can be used to fix evolutionary rates or inform a prior rate distribution for divergence time estimation [6]. Molecular clocks calibrated via secondary rates have been widely implemented in the absence of independent timing information [3], but because rates of genetic change can vary considerably among organisms and genes [1], rate constancy, a fundamental assumption for secondary rate calibrations, is easily violated [2]. Secondary rates estimated for the taxa of interest (or for closely related taxa) are ideal because these rates are more likely to be similar [7], but taxon-specific molecular clocks are unavailable for most organisms and “universal” rates for larger groups typically do not meet the assumption of rate constancy [8].

Collembola (or springtails) present an interesting, yet challenging, case of exploring evolutionary hypotheses for taxa without available node calibrations or taxon-specific molecular clocks. These minute insect-like arthropods are among the most abundant organisms in the soil mesofauna, occupy nearly all terrestrial ecosystems, and serve vital roles in detrital decomposition, food webs, and soil structure [9]. Despite their ecological importance and ubiquity in nature, springtail diversity is poorly understood, and to date, less than 2% of species are thought to have been described [10]. Cryptic species have further confounded our current understanding of springtail diversity where deeply divergent, yet morphologically conserved lineages are routinely discovered, often with molecular distances equivalent to those between genera or even families of insects [10,11,12,13,14,15,16,17,18,19,20,21,22,23]. The existence of apparent widespread cryptic diversity in springtails has driven global species richness estimates upwards of more than 500,000 species [10], but the evolutionary processes driving these patterns of diversity have not yet been explored for this group.

Patterns of morphological stasis or ‘cryptic speciation’ can be driven by directional selection on non-morphological traits such as sexual recognition, physiology, or behavior [24], and/or stabilizing selection resulting from extreme, and possibly invariant, selection pressures [25], but it remains unclear as to why the discordance between genetic and morphological variation is so prevalent in springtails. Populations may have diversified at the molecular level without morphological change over long periods of time, possibly due to invariant selection pressures on morphology [26]. The fossil record supports this ‘ancient relic’ hypothesis, with many examples of fossils being placed into extant families or genera [26,27,28,29]. Alternatively, species with highly genetically structured populations, as is common among springtails, may have an increased probability of mutations reaching fixation, which can lead to accelerated rates of molecular evolution [30]. For example, deep phylogeographic structure, large genetic distances, rapid speciation, and accelerated rates of molecular evolution have been attributed to founder effects in parasitoid wasps [31] and *Drosophila* flies [32]. Wessel et al. [33] also attributes the rapid radiation and cryptic speciation of Hawaiian cave planthoppers to this phenomenon. These studies suggest that low mobility and/or high degrees of ecological specialization, coupled with passive long-distance dispersal, may predispose some species to founder events, spatially structured populations, and ultimately, accelerated rates of molecular evolution.

Collembola share these peculiar population dynamics: they are non-vagile, with high degrees of ecological specificity, and are seemingly robust to climatic oscillations and ecological change [16]. These characteristics enable them to persist locally, often at very fine spatial scales, for long periods of time [21,34], but springtails can also passively disperse vast distances by air [35,36,37,38] and water [39,40] potentially facilitating recurrent founder events resulting in strongly structured populations. Biological factors such as shorter generation time [41], smaller effective population size [42], inefficient DNA repair mechanisms [43], smaller body size [44,45]; but see [8], asexual reproduction [46], and increased metabolic rate [44,45]; but see [47] have also been hypothesized to stimulate accelerated rates of molecular evolution. Some of these characteristics are present in springtails—some species are asexual with extremely short generation times [48], they are miniscule (body length often less than 1 mm), and they may have surprisingly small effective population sizes due to their highly structured, isolated populations.

Despite the circumstantial plausibility for accelerated molecular rates in Collembola, mitochondrial DNA clocks calibrated using rates estimated for insects, such as Papadopoulou et al.’s [4] estimate of 3.36–3.54% divergence/Ma (in tenebrionid beetles), are routinely applied in evolutionary investigations of springtail biodiversity [21,49,50,51,52,53,54,55]. Estimates of evolutionary rates in units of absolute time are essential for understanding the tempo of molecular evolution in springtails, but without independent timing information, these estimates remain dependent upon dubious secondary rate calibrations.

Nevertheless, estimating molecular rates in relative, rather than absolute, time can offer an alternative way to test evolutionary hypotheses concerning the rate of genetic change or molecular divergence for groups that lack robust calibrations. This comparative approach has been used to estimate relative divergence times for hydrobiid snails [2] and members of a solute carrier protein family [56] to determine which groups had originated most recently. Other studies have incorporated modern “relaxed” molecular clock models for estimating relative divergence times and relative rates of molecular evolution [57].

Although not within the primary scope of their study, Ciccondardi et al. [16] estimated relative rates of substitution among groups of hexapods to identify an appropriate clock calibration rate used to infer divergence times in Mediterranean springtails. To my knowledge, this is the only attempt to explicitly test rate constancy prior to applying a secondary rate clock calibration. However, methodological shortcomings limit the confidence and applicability of their results: (1) they used cytochrome oxidase subunit II (COII), a gene less commonly used for springtail phylogenetics; (2) their taxonomic coverage of Hexapoda was incomplete (98 taxa for 3 of 4 hexapod classes, including 15 of 28 insect orders); (3) they did not test for saturation, which may have impacted rate estimation; (4) phylogenetic relationships among classes and orders were not constrained (likely because many of these relationships remained unresolved at this time [58,59]), which may have produced incorrect tree topologies (not reported) that could have affected their rate estimates; (5) they did not report the model of sequence evolution used, but it was likely inferred using maximum likelihood methods due to the unavailability of Bayesian substitution model averaging methods at this time; and lastly, (6) they did not provide adequate summaries of their findings (only graphical representations of mean rates and standard error) which lack 95% highest posterior density (HPD) values—an important credibility interval for assessing significance within a Bayesian framework [60].

To expand upon the work of Cicconardi et al. [16] I use the cytochrome c oxidase I (COI) gene, rather than COII, to estimate relative rates of substitution in Hexapoda. COI is the most widely implemented gene used for secondary rate calibrations in springtails (and most other invertebrates) due to the popularity of its barcoding region, making it an ideal candidate gene for evaluating relative rates in hexapods. I perform a Bayesian phylogenetic analysis using COI sequences for 188 taxa representing every hexapod class and insect order, a constrained tree topology based on robust hexapod relationships that were independently inferred with genomic data [61], and an uncorrelated log normal “relaxed” clock model set to a mean rate of 1 substitutions/site/time to allow branch rates to vary (with respect to each other and relative to 1). This approach allowed me to avoid violating assumptions inherent with the use of secondary rate calibrations (i.e., rate constancy) or node calibrations (e.g., timing of biogeographic event), while still addressing the basic question of whether or not Collembola have accelerated rates of substitution (compared to other hexapods) and ultimately, whether or not the use of secondary rates estimated for insects are appropriate for inferring evolutionary timescales in this group.

## 2. Materials and Methods

### 2.1. Sequence Acquisition and Alignment

Complete COI sequences for a total of 204 taxa were acquired from NCBI GenBank [62] (Table S1): 188 taxa representing all classes within Hexapoda (Collembola, Protura, Diplura and all 28 insect orders) and 16 arthropod outgroup taxa. Taxa were chosen to correspond to species used by Misof et al. [61] to topologically constrain the Bayesian analysis (see below). If COI was not available for a given taxon, COI sequences for a closely related taxon were used instead (i.e., from the same genus, or same subfamily, etc.). Sequences were haphazardly chosen if multiple complete COI sequences were available in GenBank for a given taxon. For some groups (e.g., Collembola) additional taxa were included if available. All nucleotide sequences were then aligned by amino acids using MAFFT [63] implemented in TranslatorX [64]. The GBlocks [65] option in TranslatorX was used to analyze and remove columns with ambiguous homology from the nucleotide alignment based on the amino acid alignment.

### 2.2. Assessment of Substitution Saturation and Its Impact on Phylogenetic Estimation

Substitution saturation is a major concern when estimating phylogenetic parameters across deep evolutionary time [66], especially when using faster evolving markers typically used to estimate species level relationships (e.g., COI). Therefore, to make sure COI is an appropriate marker to evaluate relative substitution rates within groups of arthropods, some of which include lineages that have been independently evolving for nearly 300 million years [61], two different approaches were used to test for substitution saturation in the COI sequence alignment (1491 bp for 204 arthropod taxa). First, linear regression analyses were performed to test the linearity of relationships between uncorrected genetic distances (uncorrected p-distances) and genetic distances corrected with a general time reversible (GTR) model of sequence evolution (model-corrected p-distances). The coefficient of determination of linear regression through the origin (*R*^2^) was computed independently for each codon position. To determine if the inclusion of codon 3 would impair phylogenetic estimation, *R*^2^ for codons 1 and 2 combined was compared with the *R^2^* for all codon positions combined. If the relationship between uncorrected and model-corrected p-distances is approximately linear, then there is no saturation. If saturation is present, the relationship will deviate and begin to plateau because uncorrected p-distances will be underestimating the number of substitutions between taxa due to a loss of information after multiple substitutions at single sites [66]. At these evolutionary time scales, the third codon position for COI is likely to exhibit severe substitution saturation due to increased numbers of synonymous mutations. However, synonymous mutations should conform better to neutral theory of molecular evolution [67], and therefore, their inclusion may benefit phylogenetic estimation [68]. All uncorrected and corrected distances were calculated in PAUP* v. 4.0a build 161 [69]. 

Substitution saturation was also assessed for COI (all codons) using the substitution saturation test developed by Xia et al. [70] with DAMBE v. 6.0.0 [71]. This test evaluates whether the observed Iss (simple index of substitution saturation) is significantly lower than Iss.c (critical Iss value) derived from simulation studies [70] assuming symmetrical (Sym) and asymmetrical (Asym) tree topologies [67]. It uses a heuristic approach to randomly sample different subsets of 4, 8, 16, 32 OTUs (NumOTU) multiple times (100 jackknife replicates) to test for the presence of substitution saturation for each subset. Substitution saturation can be rejected if Iss is significantly lower than Iss.c (*p* < 0.5). The mean proportion of invariant sites used for this test (mean = 0.21) was determined via Bayesian phylogenetic analysis.

### 2.3. Relative Rate Estimation

To estimate relative rates of COI nucleotide substitution across Hexapoda I performed a Bayesian phylogenetic analysis using a fixed tree topology (Figure S1) and an uncorrelated log normal “relaxed” clock model with a mean rate equal to 1 substitution/site/time. A fixed tree topology allows us to mitigate the effect of COI’s weak resolving power for deeper nodes. Fortunately, Misof et al.’s [61] phylogenomic study produced a highly resolved and well-supported hexapod phylogeny that can be used to constrain the tree topology. Relationships from Misof et al. [61] with >98% bootstrap support were constrained to be monophyletic, while those with weaker support were collapsed to allow them to be estimated in this analysis (Figure S1). An uncorrelated log normal clock model with a mean rate set to 1 was used to allow rates to vary among branches relative to 1 substitution/site/time for all branch rates, enabling the identification of groups with faster or slower rates of substitution relative to other hexapod groups.

The Bayesian phylogenetic analysis was performed using BEAST2 v. 2.4.8 [72] with the following parameters: bModelTest [73] for site model averaging to accommodate uncertainty in the model of sequence evolution (default parameters); an uncorrelated log normal clock to allow rate variation among branches; clock.rate set to 1 to estimate branch lengths relative to 1 substitution/site/time; Yule tree prior; topological constraint prior to fix tree topology for well-supported relationships (>98% bootstrap support) inferred by Misof et al. [61] (Figure S1); MCMC for 300 million generations; sampling trees and statistics every 5000 generations; all other parameters were left as default. After applying a 10% burn-in, the effective sample size (ESS) for all parameters were determined to be greater than 200 with Tracer v. 1.7.1 [74], a total of 54,001 trees were sampled for analysis, and a maximum clade credibility tree with median node heights was inferred with TreeAnnotator v. 2.4.8 [72]. Rate summary statistics for each hexapod group were extracted from the BEAST2 output log file using TreeStat v. 1.8.4 of the BEAST v. 1.8.4 software package [75]. Significant differences between mean relative rates among hexapod groups were assessed by comparing 95% highest posterior density (HPD) rate intervals.

## 3. Results

### 3.1. Substitution Saturation Tests

**Figure 1:**
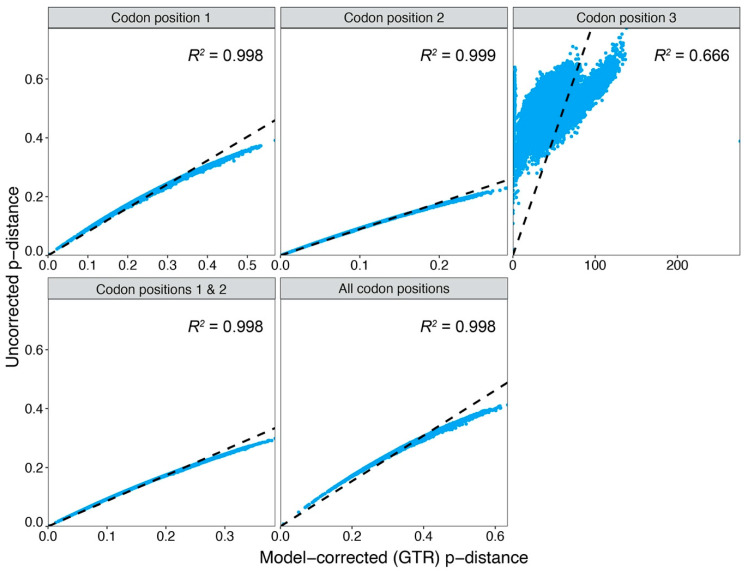
Substitution saturation plots showing the linear relationship between uncorrected and model-corrected (GTR) genetic distances for cytochrome oxidase subunit 1 (COI) codon position 1–3, codon positions 1 and 2 combine, and all codon positions combined. The linear regression (dotted line) and coefficient of determination (*R*^2^) are indicated for each plot.

Simple linear regression plots of genetic distances (uncorrected vs. model corrected) (Figure 1) identified significant substitution saturation for COI codon position 3 only (*R*^2^ = 0.666). Plots for codon 1, codon 2, and codons 1 and 2 combined, indicate strong linear correlations between uncorrected and model-corrected genetic distances (*R*^2^ = 0.998, 0.999, and 0.998, respectively). When codon 3 was combined with codons 1 and 2, the coefficient of determination remained high (*R*^2^ = 0.998), supporting the inclusion of codon 3 for estimating evolutionary rates.

Results from Xia et al.’s [70] test for substitution saturation provided an additional measure of support to justify the inclusion of codon 3 for the relative rate analysis. Iss (simple index of substitution saturation) was significantly lower than Iss.c (critical Iss value) under the assumption of both symmetrical and asymmetrical tree topologies for all OTU subsets (*p* < 0.01) (Table 1), indicating that the hypothesis of significant substitution saturation in the combined (codons 1–3) sequence alignment can be rejected. All three codons were incorporated in subsequent analyses because these results suggest that phylogenetic inferences will not be strongly impacted by substitution saturation despite the inclusion of codon 3.

### 3.2. Relative Rates of COI Substitution in Hexapoda

**Figure 2:**
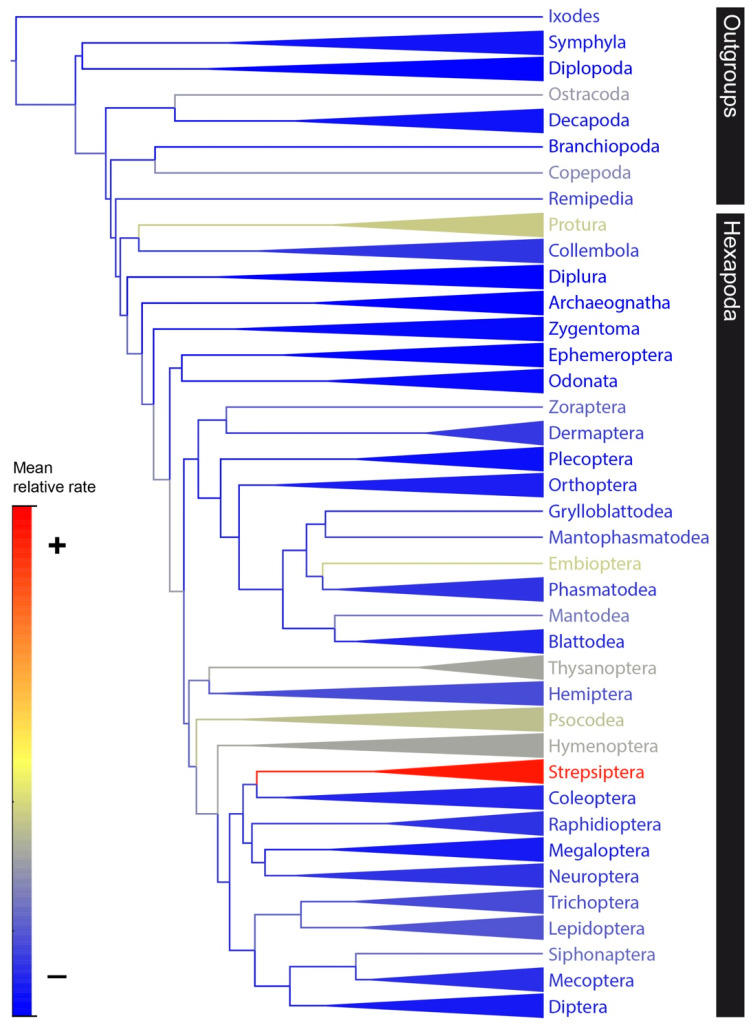
Bayesian COI gene tree using a topological constraint matching Misof et al.’s [61] phylogeny and a relaxed molecular clock set to 1 substitutions/site/time to estimate relative rates of nucleotide substitution across Hexapoda. Branch/label colors indicate mean relative rates for each clade (see scale). Clades with multiple taxa are collapsed into cones. See Figure S2 for phylogeny displaying all branches with support values for non-constrained nodes.

**Figure 3:**
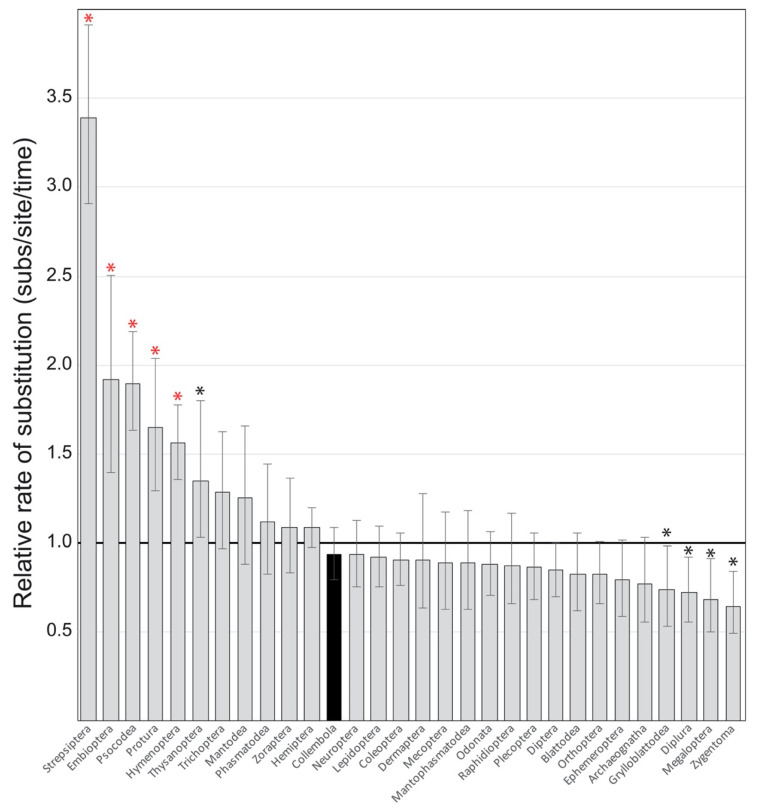
Bar chart of mean relative rates of nucleotide substitution in COI for all major Hexapod groups. Error bars represent the 95% highest posterior density (HPD) mean rate interval. Asterisks indicate rates that are significantly different from the mean rate of 1 (horizontal black line); red asterisks indicate rates that are significantly different from Collembola rates (black bar).

The Bayesian phylogenetic analysis of 204 arthropod COI sequences revealed significant variation in relative rates of nucleotide substitution among hexapod groups (Figure 2 and Figure 3, Figure S2; Table S2). Visual inspection of the maximum clade credibility tree (with branches colored to indicate rate) revealed six groups (i.e., Protura, Embioptera, Thysanoptera, Psocodea, Hymenoptera, and Strepsiptera) with greatly elevated rates of nucleotide substitution compared to other Hexapods (Figure 2, Figure S2). Overall, mean relative rates for each group ranged from 0.64 to 3.40 substitutions/site/time (Figure 3; Table S2). Ten groups had 95% HPD rate intervals that did not include 1 substitutions/site/time (mean tree rate)—Strepsiptera, Embioptera, Psocodea, Protura, Hymenoptera, and Thysanoptera were higher, while Grylloblattodea, Diplura, Megaloptera, and Zygentoma were lower. Ninety-five percent HPD mean rate intervals for all other taxonomic groups, including Collembola, spanned 1 substitutions/site/time (Figure 3; Table S2). When compared to Collembola, only five groups had significantly different rates of nucleotide substitution: Strepsiptera, Embioptera, Psocodea, Protura, and Hymenoptera (Figure 3). 

## 4. Discussion

Saturation tests indicated that the phylogenetic analysis would not be significantly impacted by substitution saturation (Table 1; Figure 1). This was relatively surprising given the taxonomic depth of the samples (i.e., Hexapoda + outgroups). It is possible that the comprehensive sampling approach, which incorporated most major lineages of Hexapoda, provided sufficient coverage of nucleotide diversity needed to facilitate accurate approximation of multiple substitutions in COI [76]. Nonetheless, only the within-clade branches (more recent/less saturated) for each group were used for rate comparisons, while the rates associated with deeper branches connecting groups (older/more saturated) were ignored.

The comparative rate analysis (Figure 2 and Figure 3; Table S2) revealed that Collembola exhibit rates within the range of rate variation for most other hexapod groups. This suggests that the application of secondary insect rate calibrations may be appropriate for estimating divergence times in springtails. Relative rates estimated in this study were also remarkably consistent with those estimated by Cicconardi et al. [16], despite differences in methodology and gene choice. Although most hexapod groups were determined to have relatively similar rates of nucleotide substitution, five groups were identified as having significantly higher rates compared to springtails (i.e., Strepsiptera, Embioptera, Psocodea, Protura, and Hymenoptera), supporting critics of secondary rate calibrations [8] and those that stress the importance of taxon-specific clocks [7]. It is also noteworthy that accelerated rates have been previously documented for most of these groups [31,77,78,79], signifying that, despite the low precision associated with estimates from this study, this method still produced accurate results that are neither unique nor unusual. In some groups there is a relatively high prevalence of parasitism (i.e., Strepsiptera, Psocodea, and Hymenoptera)—a life history trait thought to be linked to founder effects and strongly structured populations that can lead to accelerated evolutionary rates [31]. Other studies suggest that high rates of substitution in Thysanoptera, Psocodea, and Embioptera are related to frequent gene rearrangements [80,81]. The high rates for Protura were more perplexing. Protura and Collembola are phylogenetic sister taxa that share many similarities in their biology—they are both minute, non-vagile, and members of the soil mesofauna. In addition, large genetic distances between presumably conspecific taxa are commonly detected in both groups [20,82], suggesting they have similar patterns of extensive cryptic diversity. Despite these similarities, Protura substitution rates are 1.5–2 times higher than those observed for Collembola.

Because springtail substitution rates were similar to most other hexapods, their apparent morphological-molecular disparity cannot be explained by accelerated rates of molecular evolution and is instead more consistent with the hypothesis of long-term morphological stasis. However, given the limited taxonomic sampling (average rates of clades may be biased) and low precision in the rate estimates, these findings should be considered with caution, especially for hypotheses concerning mechanisms driving cryptic diversity within Collembola. Nevertheless, this study serves as a useful starting point towards understanding the rate of molecular evolution in springtails with respect to patterns of cryptic diversity and provides limited validation of previous studies that have used molecular clocks calibrated with secondary rates estimated for Coleoptera [4] to infer divergence times in springtails [21,49,50,51,52,53,54,55], as they share similar rates of nucleotide substitution (Figure 3). More importantly, this analysis exemplifies a relatively simple approach for evaluating rate constancy within a taxonomic group to determine whether the use of secondary rates are appropriate for molecular clock calibrations—a practice that is becoming increasingly scrutinized [83], despite being essential for addressing evolutionary hypotheses in groups lacking fossils or other independent timing information.

## Figures and Tables

**Table 1:** Results output for the substitution saturation test for COI implemented in DAMBE. NumOTU = OTU subset number, Iss = simple index of substitution saturation, Iss.cSym = critical index of substitution saturation assuming symmetrical tree topology, Iss.cAsym = critical index of substitution saturation assuming asymmetrical tree topology, T = *t*-value, DF = degrees of freedom, P = *p*-value.

NumOTU	Iss	Iss.cSym	T	DF	P	Iss.cAsym	T	DF	P
4	0.43	0.83	24.97	1177	0.000	0.80	23.08	1177	0.000
8	0.43	0.81	21.13	1177	0.000	0.71	16.23	1177	0.000
16	0.44	0.79	20.35	1177	0.000	0.61	9.67	1177	0.000
32	0.44	0.77	19.30	1177	0.000	0.49	2.78	1177	0.006

## Supplementary Materials

The following are available online at https://www.mdpi.com/2073-4425/11/10/1172/s1, Figure S1: Constraint tree modeled after Misof et al. [61] and used to fix tree topology for Bayesian phylogenetic analysis, Figure S2: Bayesian phylogeny (ungrouped) used to estimate relative rates of COI substitution for Hexapoda. Table S1: All taxa used in this study with corresponding GenBank accession numbers, Table S2: Relative rate summary statistics for each hexapod group compared in this study.

## References

[1] Ho S.Y.W., Duchêne S. (2014). Molecular-clock methods for estimating evolutionary rates and timescales. Mol. Ecol..

[2] Wilke T., Schultheiß R., Albrecht C. (2009). As time goes by: A simple fool’s guide to molecular clock approaches in invertebrates. Am. Malacol. Bull..

[3] Hipsley C.A., Müller J. (2014). Beyond fossil calibrations: Realities of molecular clock practices in evolutionary biology. Front. Genet..

[4] Papadopoulou A., Anastasiou I., Vogler A.P. (2010). Revisiting the insect mitochondrial molecular clock: The mid-aegean trench calibration. Mol. Biol. Evol..

[5] Ho S.Y.W., Tong K.J., Foster C.S.P., Ritchie A.M., Lo N., Crisp M.D. (2015). Biogeographic calibrations for the molecular clock. Biol. Lett..

[6] Marshall D.C., Hill K.B.R., Moulds M., Vanderpool D., Cooley J.R., Mohagan A.B., Simon C. (2016). Inflation of molecular clock rates and dates: Molecular phylogenetics, biogeography, and diversification of a global cicada radiation from Australasia (Hemiptera: Cicadidae: Cicadettini). Syst. Biol..

[7] Weir J.T., Schluter D. (2008). Calibrating the avian molecular clock. Mol. Ecol..

[8] Thomas J.A., Welch J.J., Woolfit M., Bromham L. (2006). There is no universal molecular clock for invertebrates, but rate variation does not scale with body size. Proc. Natl. Acad. Sci. USA.

[9] Hopkin S.P. (1997). Biology of Springtails (Insecta: Collembola).

[10] Cicconardi F., Fanciulli P.P., Emerson B.C. (2013). Collembola, the biological species concept and the underestimation of global species richness. Mol. Ecol..

[11] Frati F., Carapelli A., Fanciulli P.P. (1995). The genus *Isotomurus*: Where molecular markers help to evaluate the importance of morphological characters for the diagnosis of species. Pol. Pismo Entomol..

[12] Frati F., Dell’Ampio E., Casasanta S., Carapelli A., Fanciulli P.P. (2000). Large amounts of genetic divergence among Italian species of the genus *Orchesella* (Insecta, Collembola) and the relationships of two new species. Mol. Phylogenet. Evol..

[13] Soto-Adames F.N. (2002). Molecular phylogeny of the Puerto Rican *Lepidocyrtus* and *Pseudosinella* (Hexapoda: Collembola), a validation of Yoshii’s “color pattern species”. Mol. Phylogenet. Evol..

[14] Hogg I.D., Hebert P.D.N. (2004). Biological identification of springtails (Hexapoda: Collembola) from the Canadian Arctic, using mitochondrial DNA barcodes. Can. J. Zool..

[15] Timmermans M.J.T.N., Ellers J., Mariën J., Verhoef S.C., Ferwerda E.B., van Straalen N.M. (2005). Genetic structure in *Orchesella cincta* (Collembola): Strong subdivision of European populations inferred from mtDNA and AFLP markers. Mol. Ecol..

[16] Cicconardi F., Nardi F., Emerson B.C., Frati F., Fanciulli P.P. (2010). Deep phylogeographic divisions and long-term persistence of forest invertebrates (Hexapoda: Collembola) in the North-Western Mediterranean basin. Mol. Ecol..

[17] Porco D., Bedos A., Greenslade P., Janion C., Skarżyński D., Stevens M.I., Jansen van Vuuren B., Deharveng L. (2012). Challenging species delimitation in Collembola: Cryptic diversity among common springtails unveiled by DNA barcoding. Invertebr. Syst..

[18] Porco D., Potapov M., Bedos A., Busmachiu G., Weiner W.M., Hamra-Kroua S., Deharveng L. (2012). Cryptic diversity in the ubiquist species *Parisotoma notabilis* (Collembola, Isotomidae): A long-used chimeric species?. PLoS ONE.

[19] Deharveng L., Zoughailech A., Hamra-Kroua S., Porco D. (2015). A new species of *Deutonura* (Collembola: Neanuridae: Neanurinae) from north-eastern Algeria, and characterisation of two intraspecific lineages by their barcodes. Zootaxa.

[20] Katz A.D., Giordano R., Soto-Adames F.N. (2015). Operational criteria for cryptic species delimitation when evidence is limited, as exemplified by North American *Entomobrya* (Collembola: Entomobryidae). Zool. J. Linn. Soc..

[21] Katz A.D., Taylor S.J., Davis M.A. (2018). At the confluence of vicariance and dispersal: Phylogeography of cavernicolous springtails (Collembola: Arrhopalitidae, Tomoceridae) codistributed across a geologically complex karst landscape in Illinois and Missouri. Ecol. Evol..

[22] Pan Z.-X., Zhang F., Li Y.-B. (2015). Two closely related *Homidia* species (Entomobryidae, Collembola) revealed by morphological and molecular evidence. Zootaxa.

[23] Zhang F., Jantarit S., Nilsai A., Stevens M.I., Ding Y., Satasook C. (2018). Species delimitation in the morphologically conserved *Coecobrya* (Collembola: Entomobryidae): A case study integrating morphology and molecular traits to advance current taxonomy. Zool. Scr..

[24] Bickford D., Lohman D.J., Sodhi N.S., Ng P.K.L., Meier R., Winker K., Ingram K.K., Das I. (2007). Cryptic species as a window on diversity and conservation. Trends Ecol. Evol..

[25] Rothschild L.J., Mancinelli R.L. (2001). Life in extreme environments. Nature.

[26] Sánchez-García A., Engel M.S. (2016). Long-term stasis in a diverse fauna of Early Cretaceous springtails (Collembola: Symphypleona). J. Syst. Palaeontol..

[27] Mari Mutt J.A. (1983). Collembola in amber from the Dominican Republic. Proc. Entomol. Soc. Washingt..

[28] Christiansen K., Pike E. (2002). Cretaceous Collembola (Arthropoda, Hexapoda) from the Upper Cretaceous of Canada. Cretac. Res..

[29] Christiansen K., Nascimbene P. (2006). Collembola (Arthropoda, Hexapoda) from the mid Cretaceous of Myanmar (Burma). Cretac. Res..

[30] Frean M., Rainey P.B., Traulsen A. (2013). The effect of population structure on the rate of evolution. Proc. R. Soc. B Biol. Sci..

[31] Kaltenpoth M., Showers Corneli P., Dunn D.M., Weiss R.B., Strohm E., Seger J. (2012). Accelerated evolution of mitochondrial but not nuclear genomes of Hymenoptera: New evidence from crabronid wasps. PLoS ONE.

[32] DeSalle R., Templeton A.R. (1988). Founder effects and the rate of mitochondrial DNA evolution in Hawaiian *Drosophila*. Evolution.

[33] Wessel A., Hoch H., Asche M., von Rintelen T., Stelbrink B., Heck V., Stone F.D., Howarth F.G. (2013). Founder effects initiated rapid species radiation in Hawaiian cave planthoppers. Proc. Natl. Acad. Sci. USA.

[34] Garrick R.C., Rowell D.M., Simmons C.S., Hillis D.M., Sunnucks P. (2008). Fine-scale phylogeographic congruence despite demographic incongruence in two low-mobility saproxylic springtails. Evolution.

[35] Freeman J.A. (1952). Occurrence of Collembola in the air. Proc. R. Entomol. Soc. London. Ser. A Gen. Entomol..

[36] Blackith R.E., Disney R.H.L. (1988). Passive aerial dispersal during moulting in tropical Collembola. Malay. Nat. J..

[37] Coulson S.J., Hodkinson I.D., Webb N.R. (2003). Aerial dispersal of invertebrates over a high-Arctic glacier foreland: Midtre Lovénbreen, Svalbard. Polar Biol..

[38] Hawes T.C., Worland M.R., Convey P., Bale J.S. (2007). Aerial dispersal of springtails on the Antarctic Peninsula: Implications for local distribution and demography. Antarct. Sci..

[39] Hawes T.C., Worland M.R., Bale J.S., Convey P. (2008). Rafting in Antarctic Collembola. J. Zool..

[40] Coulson S.J., Hodkinson I.D., Webb N.R., Harrison J.A. (2002). Survival of terrestrial soil-dwelling arthropods on and in seawater: Implications for trans-oceanic dispersal. Funct. Ecol..

[41] Thomas J.A., Welch J.J., Lanfear R., Bromham L. (2010). A generation time effect on the rate of molecular evolution in invertebrates. Mol. Biol. Evol..

[42] Lanfear R., Kokko H., Eyre-Walker A. (2014). Population size and the rate of evolution. Trends Ecol. Evol..

[43] Britten R. (1986). Rates of DNA sequence evolution differ between taxonomic groups. Science.

[44] Martin A.P., Palumbi S.R. (1993). Body size, metabolic rate, generation time, and the molecular clock. Proc. Natl. Acad. Sci. USA.

[45] Gillooly J.F., Allen A.P., West G.B., Brown J.H. (2005). The rate of DNA evolution: Effects of body size and temperature on the molecular clock. Proc. Natl. Acad. Sci. USA.

[46] Neiman M., Hehman G., Miller J.T., Logsdon J.M., Taylor D.R. (2010). Accelerated mutation accumulation in asexual lineages of a freshwater snail. Mol. Biol. Evol..

[47] Lanfear R., Thomas J.A., Welch J.J., Brey T., Bromham L. (2007). Metabolic rate does not calibrate the molecular clock. Proc. Natl. Acad. Sci. USA.

[48] Moore J.C., Saunders P., Selby G., Horton H., Chelius M.K., Chapman A., Horrocks R.D. (2005). The distribution and life history of *Arrhopalites caecus* (Tullberg): Order: Collembola, in Wind Cave, South Dakota, USA. J. Cave Karst Stud..

[49] Zhang F., Yu D., Luo Y., Ho S.Y.W., Wang B., Zhu C. (2014). Cryptic diversity, diversification and vicariance in two species complexes of *Tomocerus* (Collembola, Tomoceridae) from China. Zool. Scr..

[50] Ding Y.-H., Yu D.-Y., Guo W.-B., Li J.-N., Zhang F. (2018). Molecular phylogeny of *Entomobrya* (Collembola: Entomobryidae) from China: Color pattern groups and multiple origins. Insect Sci..

[51] Sun X., Bedos A., Deharveng L. (2018). Unusually low genetic divergence at COI barcode locus between two species of intertidal *Thalassaphorura* (Collembola: Onychiuridae). PeerJ.

[52] Zhang F., Yu D., Stevens M.I., Ding Y. (2018). Colouration, chaetotaxy and molecular data provide species-level resolution in a species complex of *Dicranocentrus* (Collembola: Entomobryidae). Invertebr. Syst..

[53] Carapelli A., Greenslade P., Nardi F., Leo C., Convey P., Frati F., Fanciulli P.P. (2020). Evidence for cryptic diversity in the “Pan-Antarctic” springtail *Friesea antarctica* and the description of two new species. Insects.

[54] Lukić M., Delić T., Pavlek M., Deharveng L., Zagmajster M. (2020). Distribution pattern and radiation of the European subterranean genus *Verhoeffiella* (Collembola, Entomobryidae). Zool. Scr..

[55] Collins G.E., Hogg I.D., Convey P., Sancho L.G., Cowan D.A., Lyons W.B., Adams B.J., Wall D.H., Green T.G.A. (2020). Genetic diversity of soil invertebrates corroborates timing estimates for past collapses of the West Antarctic Ice Sheet. Proc. Natl. Acad. Sci. USA.

[56] Geyer J., Wilke T., Petzinger E. (2006). The solute carrier family SLC10: More than a family of bile acid transporters regarding function and phylogenetic relationships. Naunyn. Schmiedebergs. Arch. Pharmacol..

[57] Cabezas-Cruz A., Valdés J.J., Lancelot J., Pierce R.J. (2015). Fast evolutionary rates associated with functional loss in class I glucose transporters of *Schistosoma mansoni*. BMC Genom..

[58] Meusemann K., von Reumont B.M., Simon S., Roeding F., Strauss S., Kuck P., Ebersberger I., Walzl M., Pass G., Breuers S. (2010). A phylogenomic approach to resolve the arthropod tree of life. Mol. Biol. Evol..

[59] Trautwein M.D., Wiegmann B.M., Beutel R., Kjer K.M., Yeates D.K. (2012). Advances in insect phylogeny at the dawn of the postgenomic era. Annu. Rev. Entomol..

[60] Box G.E.P., Tiao G.C. (1992). Bayesian Inference in Statistical Analysis.

[61] Misof B., Liu S., Meusemann K., Peters R.S., Donath A., Mayer C., Frandsen P.B., Ware J., Flouri T., Beutel R.G. (2014). Phylogenomics resolves the timing and pattern of insect evolution. Science.

[62] Benson D.A., Cavanaugh M., Clark K., Karsch-Mizrachi I., Lipman D.J., Ostell J., Sayers E.W. (2013). GenBank. Nucleic Acids Res..

[63] Katoh K., Kuma K., Toh H., Miyata T. (2005). MAFFT version 5: Improvement in accuracy of multiple sequence alignment. Nucleic Acids Res..

[64] Abascal F., Zardoya R., Telford M.J. (2010). TranslatorX: Multiple alignment of nucleotide sequences guided by amino acid translations. Nucleic Acids Res..

[65] Castresana J. (2000). Selection of conserved blocks from multiple alignments for their use in phylogenetic analysis. Mol. Biol. Evol..

[66] Arbogast B.S., Edwards S.V., Wakeley J., Beerli P., Slowinski J.B. (2002). Estimating divergence times from molecular data on phylogenetic and population genetic timescales. Annu. Rev. Ecol. Syst..

[67] Xia X., Lemey P., Lemey P., Salemi M., Vandamme A.-M. (2009). Assessing substitution saturation with DAMBE. The Phylogenetic Handbook: A Practical Approach to DNA and Protein Phylogeny.

[68] Yang Z. (1996). Maximum-likelihood models for combined analyses of multiple sequence data. J. Mol. Evol..

[69] Swofford D.L. (2002). PAUP*. Phylogenetic Analysis Using Parsimony (*and Other Methods).

[70] Xia X., Xie Z., Salemi M., Chen L., Wang Y. (2003). An index of substitution saturation and its application. Mol. Phylogenet. Evol..

[71] Xia X. (2017). DAMBE6: New tools for microbial genomics, phylogenetics, and molecular evolution. J. Hered..

[72] Bouckaert R., Heled J., Kühnert D., Vaughan T., Wu C.H., Xie D., Suchard M.A., Rambaut A., Drummond A.J. (2014). BEAST 2: A software platform for Bayesian evolutionary analysis. PLoS Comput. Biol..

[73] Bouckaert R.R., Drummond A.J. (2017). bModelTest: Bayesian phylogenetic site model averaging and model comparison. BMC Evol. Biol..

[74] Rambaut A., Drummond A.J., Xie D., Baele G., Suchard M.A. (2018). Posterior summarization in Bayesian phylogenetics using Tracer 1.7. Syst. Biol..

[75] Drummond A.J., Suchard M.A., Xie D., Rambaut A. (2012). Bayesian phylogenetics with BEAUti and the BEAST 1.7. Mol. Biol. Evol..

[76] Philippe H., Brinkmann H., Lavrov D.V., Littlewood D.T.J., Manuel M., Wörheide G., Baurain D. (2011). Resolving difficult phylogenetic questions: Why more sequences are not enough. PLoS Biol..

[77] Hafner M.S., Sudman P.D., Villablanca F.X., Spradling T.A., Demastes J.W., Nadler S.A. (2014). Disparate rates of molecular evolution in cospeciating hosts and parasites. Science.

[78] Johnson K.P., Cruickshank R.H., Adams R.J., Smith V.S., Page R.D.M., Clayton D.H. (2003). Dramatically elevated rate of mitochondrial substitution in lice (Insecta: Phthiraptera). Mol. Phylogenet. Evol..

[79] Chen L., Chen P.-Y., Xue X.-F., Hua H.-Q., Li Y.-X., Zhang F., Wei S.-J. (2018). Extensive gene rearrangements in the mitochondrial genomes of two egg parasitoids, *Trichogramma japonicum* and *Trichogramma ostriniae* (Hymenoptera: Chalcidoidea: Trichogrammatidae). Sci. Rep..

[80] Shao R., Dowton M., Murrell A., Barker S.C. (2003). Rates of gene rearrangement and nucleotide substitution are correlated in the mitochondrial genomes of insects. Mol. Biol. Evol..

[81] Kômoto N., Yukuhiro K., Tomita S. (2012). Novel gene rearrangements in the mitochondrial genome of a webspinner, *Aposthonia japonica* (Insecta: Embioptera). Genome.

[82] Resch M.C., Shrubovych J., Bartel D., Szucsich N.U., Timelthaler G., Bu Y., Walzl M., Pass G. (2014). Where taxonomy based on subtle morphological differences is perfectly mirrored by huge genetic distances: DNA barcoding in Protura (Hexapoda). PLoS ONE.

[83] Coppard S.E., Lessios H.A. (2017). Phylogeography of the sand dollar genus *Encope*: Implications regarding the Central American Isthmus and rates of molecular evolution. Sci. Rep..

