# Supplementary material for: Inferring Evolutionary Timescales without Independent Timing Information: An Assessment of “Universal” Insect Rates to Calibrate a Collembola (Hexapoda) Molecular Clock
Source: Genes (Basel). 2020 Oct 7;11(10):1172. doi: 10.3390/genes11101172 (PMC7600954; doi:10.3390/genes11101172)

Supporting information

Table S1. All taxa used in this study with corresponding GenBank accession numbers.

Subphylum	Class/Subclass	Order	Family	Species	GenBank #
Chelicerata	Arachnida	Ixodida	Ixodidae	*Ixodes pavlovskyi*	NC_023831
Crustacea	Branchiopoda	Cladocera	Daphniidae	*Daphnia pulex*	NC_000844
Crustacea	Copepoda	Siphonostomatoida	Caligidae	*Lepeophtheirus salmonis*	NC_007215
Crustacea	Malacostraca	Decapoda	Ocypodidae	*Ocypode cordimanus*	NC_029725
Crustacea	Malacostraca	Decapoda	Ocypodidae	*Leptuca pugilator*	AF466700
Crustacea	Malacostraca	Decapoda	Penaeidae	*Litopenaeus vannamei*	NC_009626
Crustacea	Ostracoda	Myodocopida	Cypridinidae	*Vargula hilgendorfii*	NC_005306
Crustacea	Remipedia	Nectiopoda	Speleonectidae	*Speleonectes tulumensis*	NC_005938
Hexapoda	Collembola	Entomobryomorpha	Entomobryidae	*Entomobrya assuta*	KM610047
Hexapoda	Collembola	Entomobryomorpha	Entomobryidae	*Entomobryoides dissimilis*	KM610126
Hexapoda	Collembola	Entomobryomorpha	Entomobryidae	*Homidia socia*	KM610128
Hexapoda	Collembola	Entomobryomorpha	Entomobryidae	*Willowsia nigromaculata*	KM610130
Hexapoda	Collembola	Entomobryomorpha	Isotomidae	*Cryptopygus antarcticus*	NC_010533
Hexapoda	Collembola	Entomobryomorpha	Isotomidae	*Folsomotoma octooculata*	NC_024155
Hexapoda	Collembola	Entomobryomorpha	Lepidocyrtidae	*Pseudosinella violenta*	KM610132
Hexapoda	Collembola	Entomobryomorpha	Orchesellidae	*Orchesella villosa*	NC_010534
Hexapoda	Collembola	Entomobryomorpha	Seiridae	*Seira dowlingi*	KM610133
Hexapoda	Collembola	Poduromorpha	Hypogastruridae	*Gomphiocephalus hodgsoni*	NC_005438
Hexapoda	Collembola	Poduromorpha	Neanuridae	*Bilobella aurantiaca*	NC_011195
Hexapoda	Collembola	Poduromorpha	Neanuridae	*Friesea grisea*	NC_010535
Hexapoda	Collembola	Poduromorpha	Onychiuridae	*Onychiurus orientalis*	NC_006074
Hexapoda	Collembola	Poduromorpha	Onychiuridae	*Tetrodontophora bielanensis*	NC_002735
Hexapoda	Collembola	Poduromorpha	Poduridae	*Podura aquatica*	NC_006075
Hexapoda	Collembola	Symphypleona	Sminthuridae	*Sminthurus viridis*	NC_010536
Hexapoda	Diplura	Dicellurata	Japygidae	*Japyx solifugus*	NC_007214
Hexapoda	Diplura	Dicellurata	Japygidae	*Occasjapyx japonicus*	NC_022674
Hexapoda	Diplura	Dicellurata	Parajapygidae	*Parajapyx emeryanus*	NC_022673
Hexapoda	Diplura	Rhabdura	Campodeidae	*Campodea fragilis*	NC_008233
Hexapoda	Diplura	Rhabdura	Campodeidae	*Lepidocampa weberi*	NC_022675
Hexapoda	Diplura	Rhabdura	Octostigmatidae	*Octostigma sinensis*	NC_022672
Hexapoda	Insecta	Archaeognatha	Machilidae	*Pedetontus silvestrii*	NC_011717
Hexapoda	Insecta	Archaeognatha	Machilidae	*Petrobius brevistylis*	NC_007688
Hexapoda	Insecta	Archaeognatha	Machilidae	*Songmachilis xinxiangensis*	NC_021384
Hexapoda	Insecta	Archaeognatha	Machilidae	*Trigoniophthalmus alternatus*	NC_010532
Hexapoda	Insecta	Archaeognatha	Meinertellidae	*Nesomachilis australica*	NC_006895
Hexapoda	Insecta	Blattodea	Archotermopsidae	*Zootermopsis nevadensis*	NC_024658
Hexapoda	Insecta	Blattodea	Blaberidae	*Blaptica dubia*	NC_29224
Hexapoda	Insecta	Blattodea	Blattidae	*Periplaneta americana*	NC_016956
Hexapoda	Insecta	Blattodea	Cryptocercidae	*Cryptocercus kyebangensis*	NC_030191
Hexapoda	Insecta	Blattodea	Mastotermitidae	*Mastotermes darwiniensis*	NC_018120
Hexapoda	Insecta	Blattodea	Rhinotermitidae	*Prorhinotermes canalifrons*	KP026256
Hexapoda	Insecta	Coleoptera	Carabidae	*Carabus mirabilissimus*	NC_016469
Hexapoda	Insecta	Coleoptera	Curculionidae	*Dendroctonus terebrans*	JQ005146
Hexapoda	Insecta	Coleoptera	Gyrinidae	*Macrogyrus oblongus*	NC_13249
Hexapoda	Insecta	Coleoptera	Hydroscaphidae	*Hydroscapha granulum*	NC_012144
Hexapoda	Insecta	Coleoptera	Meloidae	*Epicauta chinensis*	NC_29192
Hexapoda	Insecta	Coleoptera	Meloidae	*Hycleus chodschenticus*	KT808466
Hexapoda	Insecta	Coleoptera	Meloidae	*Lytta caraganae*	NC_033339
Hexapoda	Insecta	Coleoptera	Ommatidae	*Tetraphalerus bruchi*	NC_011328
Hexapoda	Insecta	Coleoptera	Staphylinidae	*Aleochara* sp.	KT780622
Hexapoda	Insecta	Coleoptera	Tenebrionidae	*Tribolium castaneum*	NC_003081
Hexapoda	Insecta	Dermaptera	Anisolabididae	*Euborellia arcanum*	NC_032075
Hexapoda	Insecta	Dermaptera	Pygidicranidae	*Challia fletcheri*	NC_018538
Hexapoda	Insecta	Diptera	Bibionidae	*Bibio xanthopus*	KC192959
Hexapoda	Insecta	Diptera	Bombyliidae	*Bombylius major*	KC192961
Hexapoda	Insecta	Diptera	Chloropidae	*Thaumatomyia notata*	KC192976
Hexapoda	Insecta	Diptera	Culicidae	*Aedes aegypti*	NC_035159
Hexapoda	Insecta	Diptera	Culicidae	*Anopheles gambiae*	NC_002084
Hexapoda	Insecta	Diptera	Drosophilidae	*Drosophila melanogaster*	NC_024511
Hexapoda	Insecta	Diptera	Glossinidae	*Glossina morsitans*	KC192971
Hexapoda	Insecta	Diptera	Psychodidae	*Phlebotomus papatasi*	NC_028042
Hexapoda	Insecta	Diptera	Sarcophagidae	*Sarcophaga crassipalpis*	NC_026667
Hexapoda	Insecta	Diptera	Tephritidae	*Rhagoletis pomonella*	DQ006862
Hexapoda	Insecta	Diptera	Tipulidae	*Tipula cockerelliana*	NC_030520

Table S1 (Continued)

Hexapoda	Insecta	Diptera	Trichoceridae	*Trichocera bimacula*	NC_016169
Hexapoda	Insecta	Embioptera	Oligotomidae	*Aposthonia japonica*	AB639034
Hexapoda	Insecta	Ephemeroptera	Baetidae	*Baetis* sp.	GU936204
Hexapoda	Insecta	Ephemeroptera	Ephemeridae	*Ephemera orientalis*	NC_012645
Hexapoda	Insecta	Ephemeroptera	Heptageniidae	*Parafronurus youi*	NC_011359
Hexapoda	Insecta	Ephemeroptera	Isonychiidae	*Isonychia ignota*	HM143892
Hexapoda	Insecta	Ephemeroptera	Siphlonuridae	*Siphlonurus immanis*	NC_013822
Hexapoda	Insecta	Ephemeroptera	Vietnamellidae	*Vietnamella dabieshanensis*	HM067837
Hexapoda	Insecta	Grylloblattodea	Grylloblattidae	*Grylloblatta sculleni*	DQ241796
Hexapoda	Insecta	Hemiptera	Aleyrodidae	*Bemisia tabaci*	NC_006279
Hexapoda	Insecta	Hemiptera	Aleyrodidae	*Trialeurodes vaporariorum*	NC_006280
Hexapoda	Insecta	Hemiptera	Aphididae	*Acyrthosiphon pisum*	NC_011594
Hexapoda	Insecta	Hemiptera	Aphididae	*Aphis gossypii*	NC_024581
Hexapoda	Insecta	Hemiptera	Cicadidae	*Diceroprocta semicincta*	KM000131
Hexapoda	Insecta	Hemiptera	Cicadidae	*Gaeana maculata*	KM244671
Hexapoda	Insecta	Hemiptera	Cicadidae	*Magicicada tredecim*	KM000130
Hexapoda	Insecta	Hemiptera	Cicadidae	*Tettigades auropilosa*	KM000129
Hexapoda	Insecta	Hemiptera	Cydnidae	*Macroscytus gibbulus*	NC_012457
Hexapoda	Insecta	Hemiptera	Delphacidae	*Nilaparvata lugens*	NC_021748
Hexapoda	Insecta	Hemiptera	Miridae	*Adelphocoris fasciaticollis*	NC_023796
Hexapoda	Insecta	Hemiptera	Miridae	*Apolygus lucorum*	NC_023083
Hexapoda	Insecta	Hemiptera	Miridae	*Creontiades dilutus*	NC_030257
Hexapoda	Insecta	Hemiptera	Miridae	*Lygus hesperus*	NC_024641
Hexapoda	Insecta	Hemiptera	Nepidae	*Laccotrephes robustus*	NC_012817
Hexapoda	Insecta	Hemiptera	Nepidae	*Nepa hoffmanni*	NC_028084
Hexapoda	Insecta	Hemiptera	Pentatomidae	*Dolycoris baccarum*	NC_020373
Hexapoda	Insecta	Hemiptera	Pentatomidae	*Eurydema gebleri*	NC_027489
Hexapoda	Insecta	Hemiptera	Pentatomidae	*Graphosoma rubrolineatum*	NC_033875
Hexapoda	Insecta	Hemiptera	Pentatomidae	*Halyomorpha halys*	NC_013272
Hexapoda	Insecta	Hemiptera	Pentatomidae	*Nezara viridula*	NC_011755
Hexapoda	Insecta	Hemiptera	Peloridiidae	*Hackeriella veitchi*	NC_020309
Hexapoda	Insecta	Hemiptera	Peloridiidae	*Hemiodoecus leai*	NC_025329
Hexapoda	Insecta	Hemiptera	Peloridiidae	*Xenophyes cascus*	NC_024622
Hexapoda	Insecta	Hemiptera	Plataspidae	*Coptosoma bifaria*	NC_012449
Hexapoda	Insecta	Hemiptera	Plataspidae	*Megacopta cribraria*	NC_015342
Hexapoda	Insecta	Hemiptera	Tessaratomidae	*Eusthenes cupreus*	NC_022449
Hexapoda	Insecta	Hemiptera	Triozidae	*Bactericera cockerelli*	NC_030055
Hexapoda	Insecta	Hemiptera	Triozidae	*Paratrioza sinica*	NC_024577
Hexapoda	Insecta	Hemiptera	Urostylididae	*Urochela quadrinotata*	NC_020144
Hexapoda	Insecta	Hemiptera	Veliidae	*Paravelia conata*	KX821865
Hexapoda	Insecta	Hemiptera	Veliidae	*Platyvelia brachialis*	KX821864
Hexapoda	Insecta	Hemiptera	Veliidae	*Stridulivelia strigosa*	KX821866
Hexapoda	Insecta	Hymenoptera	Apidae	*Apis mellifera ligustica*	NC_001566
Hexapoda	Insecta	Hymenoptera	Apidae	*Bombus terrestris*	KT368150
Hexapoda	Insecta	Hymenoptera	Braconidae	*Cotesia vestalis*	NC_014272
Hexapoda	Insecta	Hymenoptera	Chrysididae	*Chrysis fulgida*	KU854924
Hexapoda	Insecta	Hymenoptera	Figitidae	*Leptopilina boulardi*	KU665622
Hexapoda	Insecta	Hymenoptera	Formicidae	*Atta laevigata*	KC346251
Hexapoda	Insecta	Hymenoptera	Orussidae	*Orussus occidentalis*	NC_012689
Hexapoda	Insecta	Hymenoptera	Pteromalidae	*Nasonia vitripennis*	EU746609
Hexapoda	Insecta	Hymenoptera	Tenthredinidae	*Tenthredo tienmushana*	KR703581
Hexapoda	Insecta	Lepidoptera	Bombycidae	*Bombyx mori*	NC_002355
Hexapoda	Insecta	Lepidoptera	Hepialidae	*Ahamus yunnanensis*	NC_018095
Hexapoda	Insecta	Lepidoptera	Hepialidae	*Endoclita signifer*	NC_029873
Hexapoda	Insecta	Lepidoptera	Hepialidae	*Napialus hunanensis*	NC_024424
Hexapoda	Insecta	Lepidoptera	Hepialidae	*Thitarodes pui*	NC_023530
Hexapoda	Insecta	Lepidoptera	Lycaenidae	*Celastrina hersilia*	NC_018049
Hexapoda	Insecta	Lepidoptera	Lycaenidae	*Cupido argiades*	NC_020779
Hexapoda	Insecta	Lepidoptera	Lycaenidae	*Shijimiaeoides divina*	NC_029763
Hexapoda	Insecta	Lepidoptera	Papilionidae	*Parides eurimedes*	AY804371
Hexapoda	Insecta	Lepidoptera	Sphingidae	*Manduca sexta*	NC_010266
Hexapoda	Insecta	Lepidoptera	Yponomeutidae	*Yponomeuta malinellus*	YMU09206
Hexapoda	Insecta	Lepidoptera	Zygaenidae	*Rhodopsona rubiginosa*	NC_025761
Hexapoda	Insecta	Mantodea	Mantidae	*Mantis religiosa*	NC_030265
Hexapoda	Insecta	Mantophasmatodea	Mantophasmatidae	*Sclerophasma paresisense*	NC_007701
Hexapoda	Insecta	Mecoptera	Bittacidae	*Bittacus pilicornis*	NC_015118
Hexapoda	Insecta	Mecoptera	Boreidae	*Boreus elegans*	NC_015119

Table S1 (Continued)

Hexapoda	Insecta	Mecoptera	Nannochoristidae	*Nannochorista philpotti*	HQ696580
Hexapoda	Insecta	Mecoptera	Panorpidae	*Neopanorpa pulchra*	NC_013180
Hexapoda	Insecta	Megaloptera	Corydalidae	*Corydalus cornutus*	NC_011276
Hexapoda	Insecta	Megaloptera	Sialidae	*Sialis hamata*	NC_013256
Hexapoda	Insecta	Neuroptera	Chrysopidae	*Apochrysa matsumurae*	NC_015095
Hexapoda	Insecta	Neuroptera	Chrysopidae	*Chrysopa pallens*	NC_019618
Hexapoda	Insecta	Neuroptera	Chrysopidae	*Chrysoperla nipponensis*	NC_015093
Hexapoda	Insecta	Neuroptera	Coniopterygidae	*Semidalis aleyrodiformis*	KT425067
Hexapoda	Insecta	Neuroptera	Myrmeleontidae	*Bullanga florida*	NC_032298
Hexapoda	Insecta	Neuroptera	Myrmeleontidae	*Epacanthaclisis banksi*	NC_025905
Hexapoda	Insecta	Neuroptera	Myrmeleontidae	*Myrmeleon immanis*	NC_024826
Hexapoda	Insecta	Neuroptera	Osmylidae	*Thyridosmylus langii*	NC_021415
Hexapoda	Insecta	Odonata	Calopterygidae	*Atrocalopteryx atrata*	NC_027181
Hexapoda	Insecta	Odonata	Calopterygidae	*Vestalis melania*	NC_023233
Hexapoda	Insecta	Odonata	Coenagrionidae	*Ischnura pumilio*	NC_021617
Hexapoda	Insecta	Odonata	Coenagrionidae	*Megaloprepus caerulatus*	NC_031823
Hexapoda	Insecta	Odonata	Epiophlebiidae	*Epiophlebia superstes*	NC_023232
Hexapoda	Insecta	Odonata	Euphaeidae	*Euphaea decorata*	NC_026058
Hexapoda	Insecta	Odonata	Gomphidae	*Davidius lunatus*	NC_012644
Hexapoda	Insecta	Odonata	Libellulidae	*Hydrobasileus croceus*	NC_025758
Hexapoda	Insecta	Odonata	Libellulidae	*Orthetrum chrysis*	NC_032048
Hexapoda	Insecta	Odonata	Platycnemididae	*Platycnemis foliacea*	NC_027180
Hexapoda	Insecta	Odonata	Pseudolestidae	*Pseudolestes mirabilis*	NC_020636
Hexapoda	Insecta	Orthoptera	Acrididae	*Chorthippus chinensis*	NC_011095
Hexapoda	Insecta	Orthoptera	Acrididae	*Euchorthippus fusigeniculatus*	NC_014449
Hexapoda	Insecta	Orthoptera	Acrididae	*Gomphocerippus rufus*	NC_014349
Hexapoda	Insecta	Orthoptera	Acrididae	*Gomphocerus licenti*	NC_013847
Hexapoda	Insecta	Orthoptera	Gryllotalpidae	*Gryllotalpa unispina*	NC_029148
Hexapoda	Insecta	Orthoptera	Rhaphidophoridae	*Diestrammena asynamora*	NC_029148
Hexapoda	Insecta	Orthoptera	Rhaphidophoridae	*Troglophilus neglectus*	NC_033989
Hexapoda	Insecta	Orthoptera	Tetrigidae	*Tetrix japonica*	NC_018543
Hexapoda	Insecta	Phasmatodea	Heteropterygidae	*Heteropteryx dilatata*	NC_014680
Hexapoda	Insecta	Phasmatodea	Heteropterygidae	*Orestes mouhotii*	AB477462
Hexapoda	Insecta	Phasmatodea	Timematidae	*Timema californicum*	DQ241799
Hexapoda	Insecta	Plecoptera	Capniidae	*Apteroperla tikumana*	NC_027698
Hexapoda	Insecta	Plecoptera	Capniidae	*Capnia zijinshana*	NC_034661
Hexapoda	Insecta	Plecoptera	Gripopterygidae	*Zelandoperla fenestrata*	NC_034997
Hexapoda	Insecta	Plecoptera	Nemouridae	*Nemoura nankinensis*	NC_034939
Hexapoda	Insecta	Plecoptera	Perlidae	*Dinocras cephalotes*	NC_022843
Hexapoda	Insecta	Plecoptera	Perlidae	*Kamimuria chungnanshana*	NC_028076
Hexapoda	Insecta	Plecoptera	Perlidae	*Acroneuria hainana*	NC_026104
Hexapoda	Insecta	Plecoptera	Pteronarcyidae	*Pteronarcella badia*	NC_029248
Hexapoda	Insecta	Plecoptera	Pteronarcyidae	*Pteronarcys princeps*	NC_006133
Hexapoda	Insecta	Plecoptera	Styloperlidae	*Styloperla spinicercia*	NC_034809
Hexapoda	Insecta	Psocodea	Boopiidae	*Heterodoxus macropus*	NC_002651
Hexapoda	Insecta	Psocodea	Liposcelididae	*Liposcelis sculptilimacula*	KX171073
Hexapoda	Insecta	Psocodea	Pediculidae	*Pediculus humanus capitis*	KC685833
Hexapoda	Insecta	Psocodea	Pediculidae	*Pediculus humanus corporis*	KC685832
Hexapoda	Insecta	Psocodea	Psocidae	*Longivalvus hyalospilus*	JQ910986
Hexapoda	Insecta	Psocodea	Trichopsocidae	*Psococerastis albimaculata*	NC_021400
Hexapoda	Insecta	Raphidioptera	Inocelliidae	*Inocellia fujiana*	KT425085
Hexapoda	Insecta	Raphidioptera	Raphidiidae	*Xanthostigma gobicola*	KT425093
Hexapoda	Insecta	Siphonaptera	Ceratophyllidae	*Jellisonia amadoi*	NC_022710
Hexapoda	Insecta	Strepsiptera	Mengenillidae	*Mengenilla moldrzyki*	NC_018545
Hexapoda	Insecta	Strepsiptera	Stylopidae	*Xenos vesparum*	AM286745
Hexapoda	Insecta	Thysanoptera	Thripidae	*Frankliniella intonsa*	NC_021378
Hexapoda	Insecta	Thysanoptera	Thripidae	*Thrips imaginis*	NC_004371
Hexapoda	Insecta	Trichoptera	Hydropsychidae	*Hydropsyche pellucidula*	NC_029246
Hexapoda	Insecta	Trichoptera	Limnephilidae	*Limnephilus decipiens*	NC_026219
Hexapoda	Insecta	Zoraptera	Zorotypidae	*Zorotypus medoensis*	NC_026077
Hexapoda	Insecta	Zygentoma	Ateluridae	*Atelura formicaria*	NC_011197
Hexapoda	Insecta	Zygentoma	Lepismatidae	*Thermobia domestica*	NC_006080
Hexapoda	Insecta	Zygentoma	Tricholepidiidae	*Tricholepidion gertschi*	NC_005437
Hexapoda	Protura	Acerentomata	Acerentomidae	*Acerella muscorum*	NC_026675
Hexapoda	Protura	Acerentomata	Acerentomidae	*Acerentomon microrhinus*	NC_026666
Myriapoda	Diplopoda	Callipodida	Abacionidae	*Abacion magnum*	NC_021932
Myriapoda	Diplopoda	Julida	Julidae	*Anaulaciulus koreanus*	NC_034656

Table S1 (Continued)

Myriapoda	Diplopoda	Julida	Nemasomatidae	*Antrokoreana gracilipes*	NC_010221
Myriapoda	Diplopoda	Platydesmida	Andrognathidae	*Brachycybe lecontii*	NC_021934
Myriapoda	Diplopoda	Polydesmida	Xystodesmidae	*Appalachioria falcifera*	NC_021933
Myriapoda	Diplopoda	Spirobolida	Spirobolidae	*Narceus annularus*	NC_003343
Myriapoda	Symphyla	—	Scolopendrellidae	*Symphylella* sp.	NC_011572
Myriapoda	Symphyla	—	Scutigerellidae	*Scutigerella causeyae*	NC_008453

Table S2. Relative rate summary statistics for each hexapod group compared in this study. Units in substitutions/site/time.

Taxonomic Group	Mean	Stderr of Mean	Stdev	Variance	Median	95% HPD
Strepsiptera	3.40	0.006	0.26	0.07	3.39	2.91–3.91
Embioptera	1.92	0.012	0.29	0.08	1.90	1.40–2.51
Psocodea	1.90	0.003	0.14	0.02	1.89	1.64–2.18
Protura	1.65	0.007	0.20	0.04	1.62	1.29–2.04
Hymenoptera	1.56	0.003	0.11	0.01	1.55	1.35–1.77
Thysanoptera	1.35	0.010	0.21	0.04	1.30	1.03–1.80
Trichoptera	1.28	0.004	0.17	0.03	1.27	0.97–1.62
Mantodea	1.25	0.007	0.20	0.04	1.23	0.88–1.66
Phasmatodea	1.12	0.006	0.16	0.03	1.10	0.82–1.44
Hemiptera	1.09	0.002	0.06	0.00	1.09	0.98–1.20
Zoraptera	1.09	0.003	0.14	0.02	1.07	0.83–1.36
Collembola	0.94	0.003	0.08	0.01	0.94	0.80–1.09
Neuroptera	0.93	0.003	0.10	0.01	0.93	0.76–1.12
Lepidoptera	0.92	0.003	0.09	0.01	0.92	0.76–1.10
Coleoptera	0.90	0.002	0.08	0.01	0.90	0.76–1.06
Dermaptera	0.90	0.007	0.18	0.03	0.86	0.63–1.28
Mantophasmatodea	0.89	0.005	0.15	0.02	0.87	0.63–1.18
Mecoptera	0.89	0.004	0.14	0.02	0.87	0.63–1.17
Odonata	0.88	0.004	0.09	0.01	0.88	0.71–1.06
Raphidioptera	0.87	0.004	0.14	0.02	0.84	0.66–1.17
Plecoptera	0.86	0.005	0.10	0.01	0.86	0.68–1.05
Diptera	0.85	0.003	0.08	0.01	0.84	0.70–1.00
Blattodea	0.83	0.004	0.11	0.01	0.82	0.62–1.05
Orthoptera	0.83	0.002	0.09	0.01	0.82	0.66–1.01
Ephemeroptera	0.79	0.005	0.11	0.01	0.78	0.59–1.02
Archaeognatha	0.77	0.008	0.13	0.02	0.75	0.55–1.04
Grylloblattodea	0.74	0.004	0.12	0.02	0.73	0.53–0.98
Diplura	0.72	0.004	0.10	0.01	0.71	0.56–0.92
Megaloptera	0.68	0.002	0.11	0.01	0.66	0.50–0.91
Zygentoma	0.64	0.003	0.10	0.01	0.62	0.49–0.84

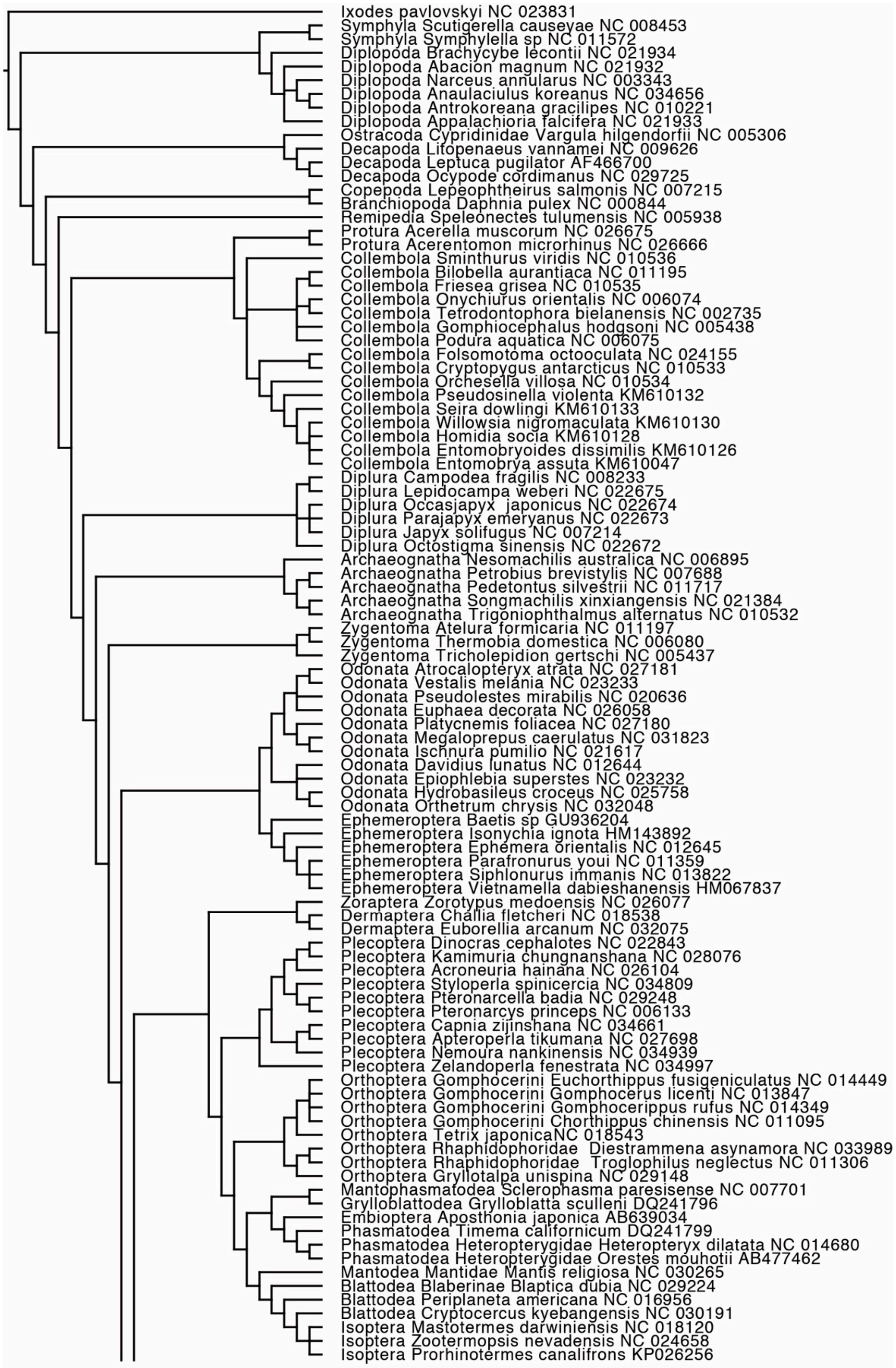

Figure S1. Constraint tree modeled after Misof *et al*. [61] and used to fix tree topology for Bayesian phylogenetic analysis.

Figure S1 (Continued)

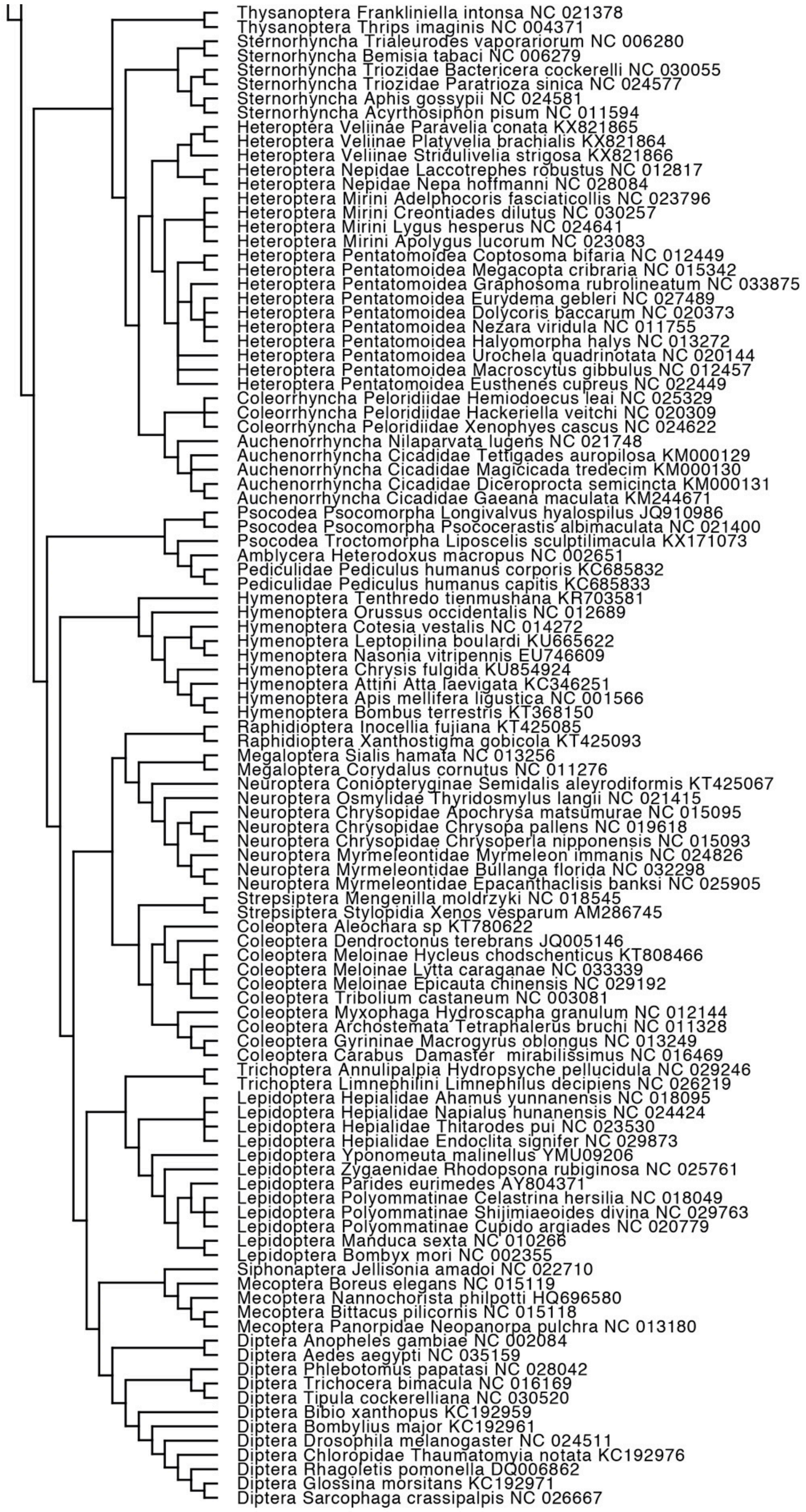

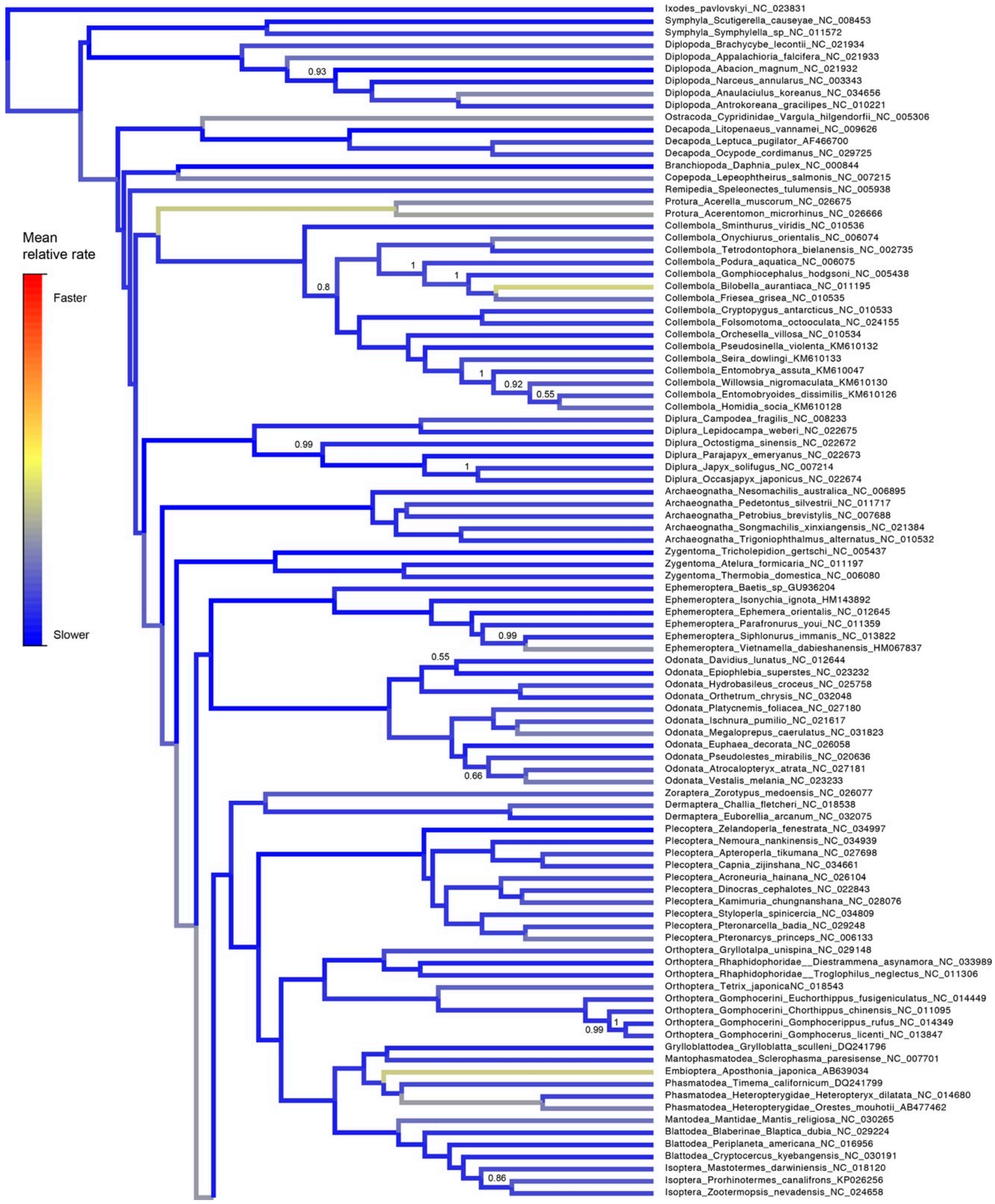

Figure S2. Bayesian phylogeny (ungrouped) used to estimate relative rates of COI substitution for Hexapoda. Branch color indicates relative rate (see legend). Unconstrained nodes have posterior probability support values. Scale bar units in substitutions/site/time.

Figure S2 (Continued)

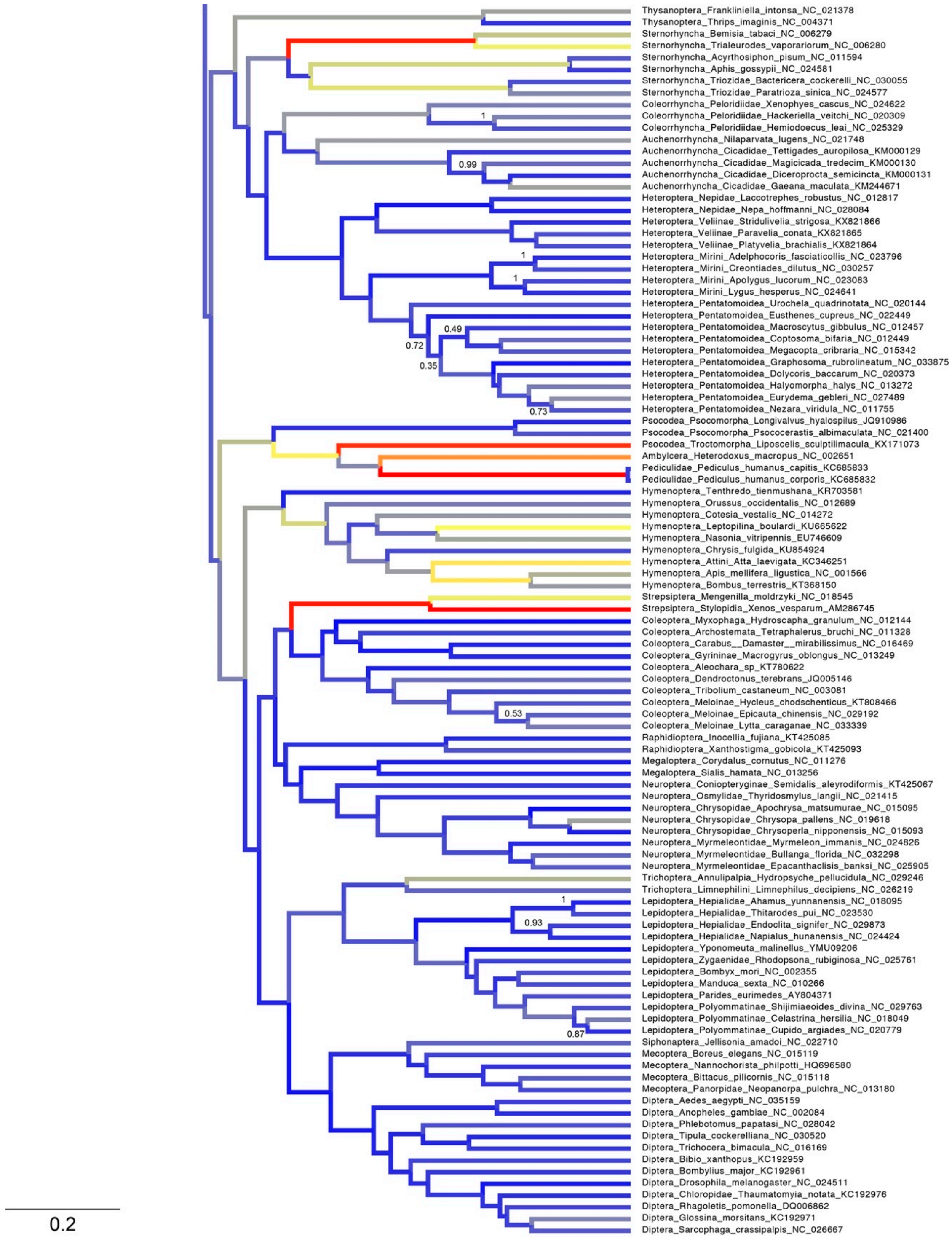

Supplement: Supplementary file 1 [file genes-11-01172-s001.pdf]
